# A tissue based chemical proteomics screen to identify novel G-kinase associated proteins (GKAPs)

**DOI:** 10.1186/2050-6511-14-S1-P16

**Published:** 2013-08-29

**Authors:** Eleonora Corradini, Pepijn P Burgers, Michael Plank, Albert JR Heck, Arjen Scholten

**Affiliations:** 1 Biomolecular Mass Spectrometry and Proteomics, Utrecht University, Utrecht, The Netherlands

## Background

Within the same cell, different signaling routes can signal through the same kinase. To still function in a specific manner, the kinase is localized in close proximity to its substrates and upstream signaling components. This is best described for cAMP-dependent protein kinase (PKA), which utilizes the diverse family of A-kinase anchoring proteins (AKAPs, >50 identified) for spatiotemporal control.

PKA’s closest homologue is the cGMP-dependent protein kinase (PKG), however many details of its localized signaling are not well understood. Thus far, very little G-kinase anchoring proteins (GKAPs) have been identified, likely because they are lower in abundance than AKAPs. In addition, unlike as for AKAPs, common motifs in GKAPs that mediate the interaction have not been convincingly defined.

## Results

Here we present a chemical proteomics approach which utilizes immobilized cAMP in combination with low concentrations of free cAMP or cGMP to specifically dissect the lower abundant PKG and its associated GKAPs, from the more prevalent PKA and interacting AKAPs. Combined with LC-MS/MS this proved a powerful screening tool for the discovery and characterization of novel protein kinase G anchoring proteins directly in several different tissues.

The isolated PKG fraction contained several known GKAPs (e.g. IRAG), but also a novel putative GKAP, Huntingtin associated protein 1 (HAP1). Further characterization of the PKG-HAP1 interaction was performed with classical biochemistry, cell biology and an interaction-based proteomics approach with immunoprecipitations of GFP-HAP1 and PKG-GFP. This powerful combination confirmed the interaction and also revealed a further refinement of the cellular localization and molecular environment of the HAP1-PKG complex.

## Conclusion

Our data highlight the power of a tailored chemical proteomics approach to identify novel cGMP signaling hubs in a tissue of interest.

